# Author Correction: Defective HNF4alpha-dependent gene expression as a driver of hepatocellular failure in alcoholic hepatitis

**DOI:** 10.1038/s41467-023-36548-3

**Published:** 2023-02-10

**Authors:** Josepmaria Argemi, Maria U. Latasa, Stephen R. Atkinson, Ilya O. Blokhin, Veronica Massey, Joel P. Gue, Joaquin Cabezas, Juan J. Lozano, Derek Van Booven, Aaron Bell, Sheng Cao, Lawrence A. Vernetti, Juan P. Arab, Meritxell Ventura-Cots, Lia R. Edmunds, Constantino Fondevila, Peter Stärkel, Laurent Dubuquoy, Alexandre Louvet, Gemma Odena, Juan L. Gomez, Tomas Aragon, Jose Altamirano, Juan Caballeria, Michael J. Jurczak, D. Lansing Taylor, Carmen Berasain, Claes Wahlestedt, Satdarshan P. Monga, Marsha Y. Morgan, Pau Sancho-Bru, Philippe Mathurin, Shinji Furuya, Carolin Lackner, Ivan Rusyn, Vijay H. Shah, Mark R. Thursz, Jelena Mann, Matias A. Avila, Ramon Bataller

**Affiliations:** 1 Division of Gastroenterology, Hepatology and Nutrition, Pittsburgh Liver Research Center, University of Pittsburgh Medical Center (UPMC), Pittsburgh, PA 15261 USA; 2 Liver Unit, Clínica Universidad de Navarra, University of Navarra, Pamplona, 31008 Spain; 3 Hepatology Program, Center for Applied Medical Research (CIMA), University of Navarra, Pamplona, 31008 Spain; 4 Division of Digestive Diseases, Department of Surgery and Cancer, Imperial College London, London, SW7 2AZ UK; 5 Center for Therapeutic Innovation and Department of Psychiatry and Behavioral Sciences, University of Miami Miller School of Medicine, Miami, FL 33136 USA; 6 Division of Gastroenterology and Hepatology, Departments of Medicine and Nutrition and Bowles Center for Alcohol Studies, University of North Carolina at Chapel Hill, Chapel Hill, NC 27516 USA; 7 Departament of Hepatology, Marqués de Valdecilla University Hospital, Santander, 39008 Spain; 8 Centro de Investigacion Biomedica en Red, Enfermedades Hepáticas y Digestivas (CIBERehd), Madrid, 28029 Spain; 9 Institut d’Investigacions Biomèdiques August Pi i Sunyer (IDIBAPS), Barcelona, 08036 Spain; 10 John P. Hussman Institute of Human Genomics. Miller School of Medicine, University of Miami, Miami, FL 33136 USA; 11 Departments of Pathology and Medicine, Pittsburgh Liver Research Center, University of Pittsburgh School of Medicine, Pittsburgh, PA 15261 USA; 12 Division of Gastroenterology and Hepatology, Mayo Clinic, Rochester, MN 55905 USA; 13 University of Pittsburgh Drug Discovery Institute, Department of Computational & Systems Biology, University of Pittsburgh, Pittsburgh, PA 15261 USA; 14 Departamento de Gastroenterologia, Escuela de Medicina, Pontificia Universidad Catolica de Chile, Santiago, Chile; 15 Department of Medicine, Division of Endocrinology and Metabolism, Center for Metabolic and Mitochondrial Medicine, University of Pittsburgh, Pittsburgh, PA 15261 USA; 16 Liver Transplant Unit, Department of Surgery, Hospital Clinic, University of Barcelona, Barcelona, 08036 Spain; 17 Service d’Hépato-gastroentérologie, Cliniques Universitaires Saint-Luc and Laboratory of Hepatogastroenterology, Institut de Recherche Expérimentale et Clinique, Université Catholique de Louvain, Brussels, 1200 Belgium; 18 Service des Maladies de l’appareil digestif, CHU Lille. Inserm LIRIC - UMR995, University of Lille, Lille, 59000 France; 19 Department of Gene Therapy and Regulation, Center for Applied Medical Research, University of Navarra, Pamplona, 31008 Spain; 20 Liver Unit, Department of Internal Medicine, Vall d’Hebron Institut de Recerca. Internal Medicine Department, Hospital Quiron Salud, Barcelona, 08035 Spain; 21 UCL Institute for Liver and Digestive Health, Division of Medicine, Royal Free Campus, University College London, London, WC1E 6BT UK; 22 Department of Veterinary Integrative Biosciences, College of Veterinary Medicine and Biomedical Sciences, Texas A&M University, College Station, TX 77845 USA; 23 Medical University of Graz, Institute of Pathology, Graz, 8036 Austria; 24 Newcastle Fibrosis Research Group, Institute of Cellular Medicine, Faculty of Medical Sciences, Newcastle University, Newcastle upon Tyne, NE2 4HH UK

Correction to: *Nature Communications* 10.1038/s41467-019-11004-3, published online 16 July 2019

This Article contains an error in the spelling of the author Constantino Fondevila, which is incorrectly given as Constantino Fondevilla. The error has been corrected in the PDF and HTML versions of the Article.

