# Supplementary material for: Defective HNF4alpha-dependent gene expression as a driver of hepatocellular failure in alcoholic hepatitis
Source: Nat Commun. 2019 Jul 16;10:3126. doi: 10.1038/s41467-019-11004-3 (PMC6635373; doi:10.1038/s41467-019-11004-3)
Supplement: Supplementary file 1 — Supplementary Information [file 41467_2019_11004_MOESM1_ESM.pdf]

“Defective HNF4alpha-dependent gene expression as a driver of hepatocellular failure in alcoholic hepatitis”

Argemi J. et al.

Supplementary Information

Supplementary Fig. 1. Schematic Work Flowchart of Human Samples analyses.
Supplementary Fig. 2. Time-dependent patterns of gene expression in ALD patients.
Supplementary Fig. 3. Transcriptome changes in ALD reflect hepatocellular disfunction.
Supplementary Fig. 4. Mouse model of acute-on-chronic alcohol mediated liver injury partially reproduces features of human AH transcription factor reprogramming.
Supplementary Fig. 5. RNA-seq analysis reveals *HNF4A* splicing dysregulation in patients with AH.
Supplementary Fig. 6. Immunohistochemistry of HNF1α and FOXA1 show decreased nuclear signal and cytoplasmic staining in AH patients.
Supplementary Fig. 7. Markers of hepatocyte de-differentiation and epithelial-to-mesenchymal transition (EMT) are increased in AH patients.
Supplementary Fig. 8. Silencing of HNF4a-P2 enhances Glyco conjugation of cheno-deoxycolic acid in primary human hepatocytes.
Supplementary Fig. 9. Mechanism of TGFβ1 and AREG-mediated HNF4α downregulation**.**
Supplementary Fig. 10. c-JUN sites in HNF4a-P2 promoter region (TSS+/-1kb)
Supplementary Fig. 11. Plasma liver-secreted LETF-dependent protein levels correlate with liver transcript changes.
Supplementary Fig. 12. Complementary approach for identifying transcription factors that regulate liver transcriptome.
Supplementary Fig. 13. Uncropped blots from main figures’ panels.

Supplementary Table 1. Patient Characteristics
Supplementary Table 2. Oligonucleotides used for PCR
Supplementary Table 3. Antibodies used for Western Blot and ChIP-PCR
Supplementary Table 4. Immunohistochemistry conditions

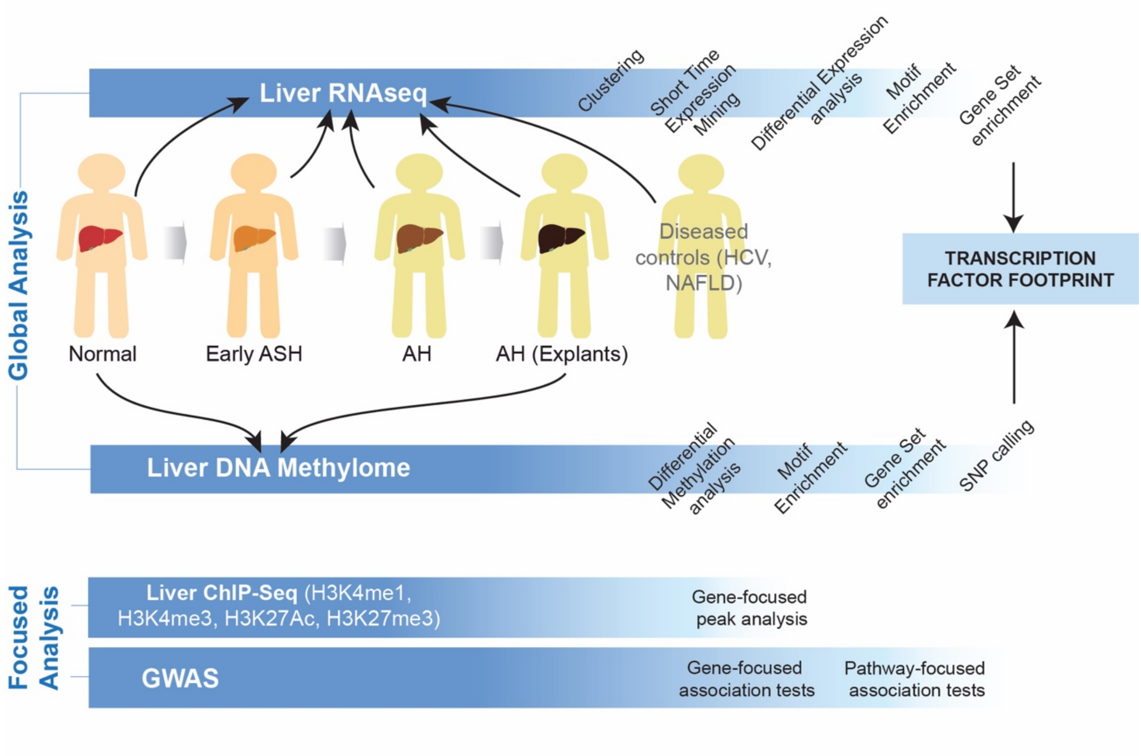

Supplementary Fig. 1. Schematic Work Flowchart of Human Samples analyses. We did two global unbiased analyses (RNA sequencing and DNA Methylation chip) and two focused studies (Liver ChIP-seq and GWAS). For details in cohorts analyzed and the type of analysis performed in this study see Materials and Methods section.

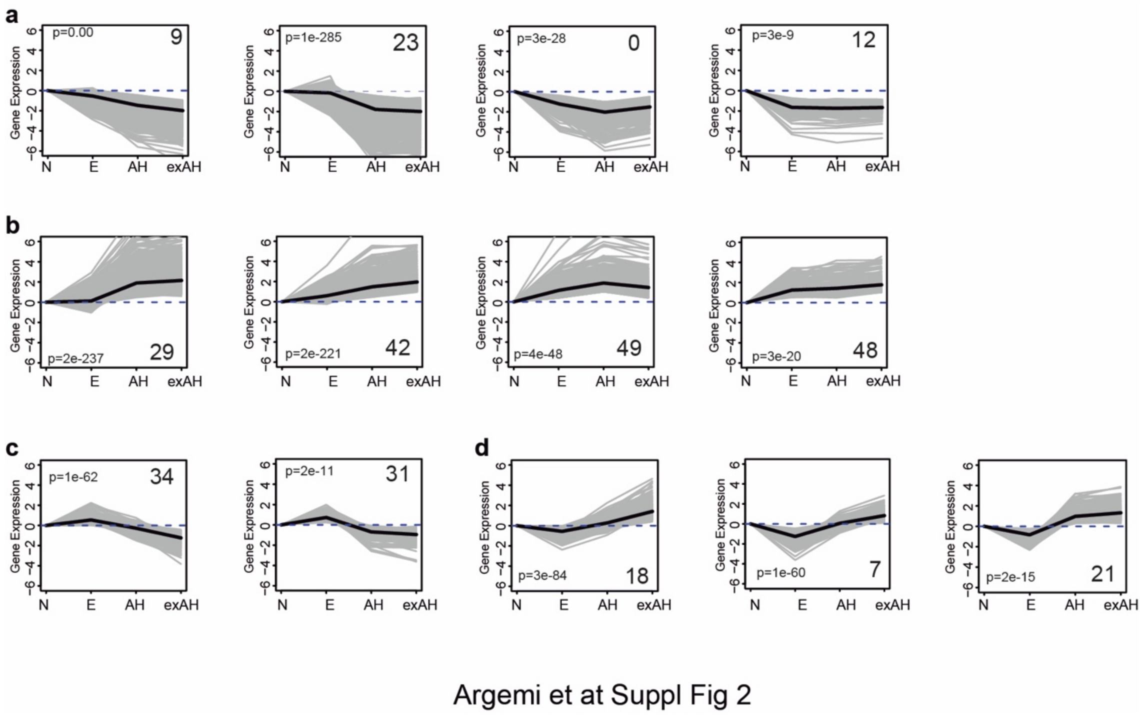

Supplementary Fig. 2. Time-dependent patterns of gene expression in ALD patients. Linear plot of normalized counts for each profile of genes derived from Short Time Expression Mining (STEM) algorithm along different phenotypes reflecting disease progression. (**a**) STEM profiles 9, 23, 0 and 12, showing patterns of continuous downregulation, (**b**) STEM profiles 29, 42, 49 and 48, showing patterns of continuous upregulation (**c**) STEM profiles 34 and 31, showing patterns of upregulation in early ASH patients and downregulation in AH patients. (**d**) STEM profiles 18, 7 and 21, showing downregulation in early ASH patients and upregulation in AH patients. P-values of profile enrichment are presented.. N: Normal Liver, E: Early Alcoholic Steato-hepatitis (ASH), AH: Alcoholic Hepatitis, exAH: explants from patients transplanted for AH.

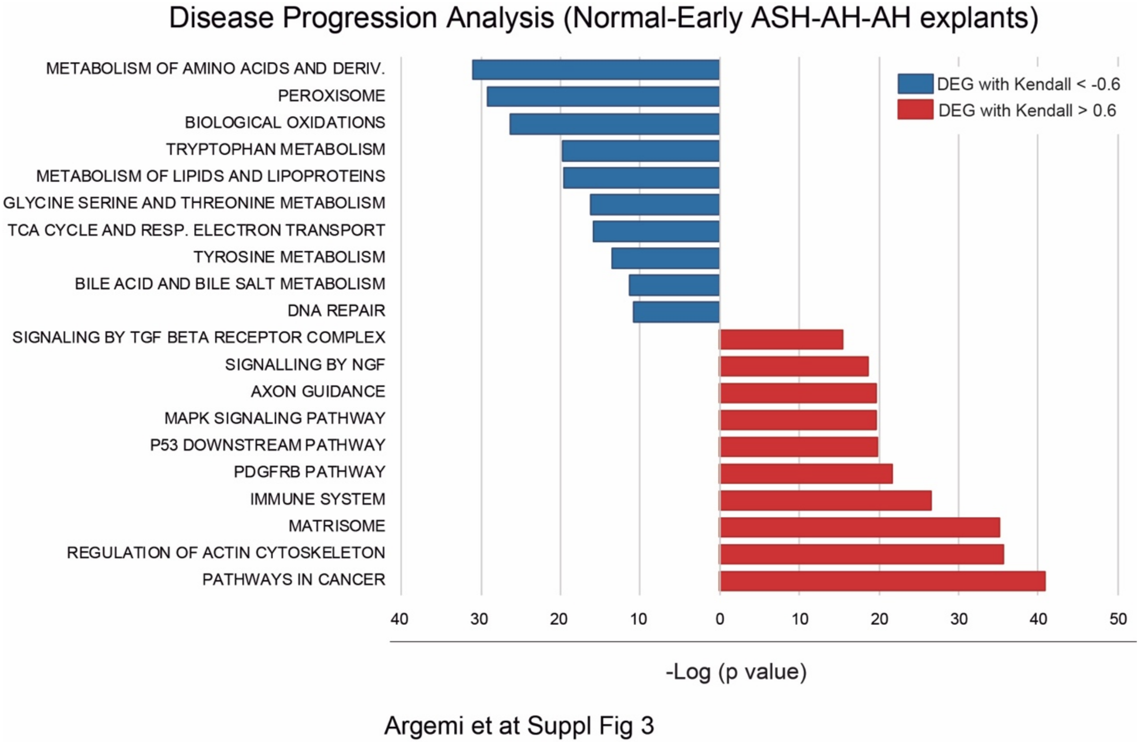

Supplementary Fig. 3. Transcriptome changes in ALD reflect hepatocellular disfunction. Results of Gene Set Expression Analysis (GSEA) performed by computing gene set overlaps of the 2000 genes most positively and negatively correlated with disease progression from normal livers to explants from livers with AH (exAH). Top 10 most enriched gene sets are shown.

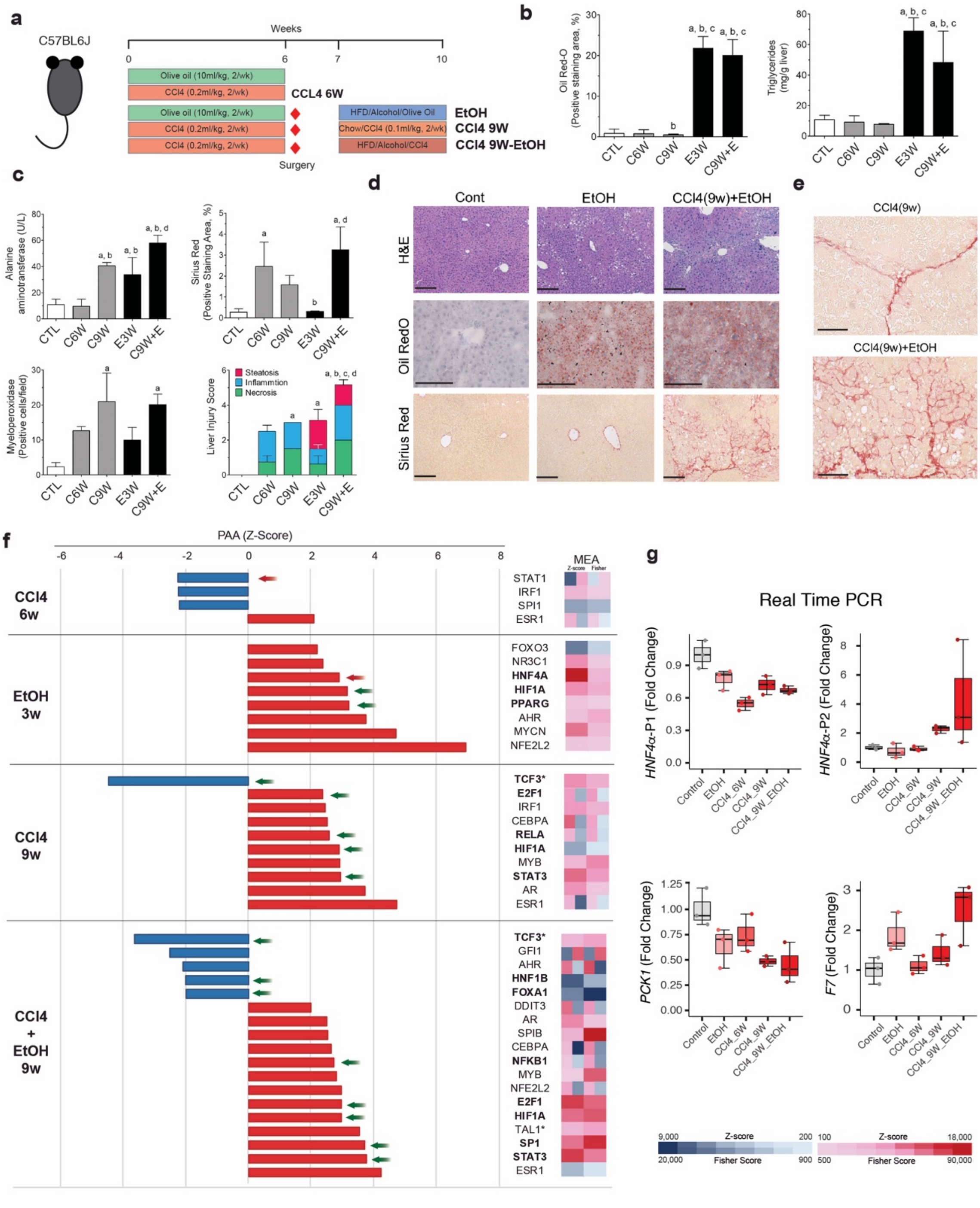

Argemi et al. Supplementary Fig. 4

Supplementary Fig. 4. Mouse model of acute-on-chronic alcohol mediated liver injury partially reproduces features of human AH transcription factor reprogramming. (a) Five experimental models were used in order to reproduce different stages and grades of alcohol-related liver injury in C57BL/6J mice. Liver RNA was extracted and sequenced (*n* = 4 per each group): **Group 1:** Olive oil for 6 weeks ("*Control*"); **Group 2:** High dose CCl_4 (0.2 ml/kg) for 6 weeks ("*CCl_4 6W*"). **Group 3:** Olive oil for 6 weeks + surgery (intra-gastric canulae) and 1 week post-op recovery + Ethanol for 3 more weeks ("*EtOH*"); **Group 4:** High dose CCl4 for 6 weeks + surgery (intra-gastric canulae) and 1 week post-op recovery + low dose CCl_4 (0.1 ml/kg) for 3 more weeks ("*CCl_4 9W*"); **Group 5:** High dose CCl4 for 6 weeks + surgery (intra-gastric canulae) and 1 week post-op recovery + Ethanol and low dose CCl_4 for 3 more weeks ("*CCl_4 9W+EtOH*") **(b-e)** Biochemical and histological analysis of liver injury **(b)** Steatosis assessment: quantitative

analysis of Oil RedO staining (5 random fields at 200× magnification) and triglyceride levels in liver tissue. **(c)** Inflammation and fibrosis assessment: Serum alanine aminotransferase levels and quantitative analysis of MPO-positive cell counts and Sirius red staining (5 random fields at 200× magnification). A composite Liver Injury Score was calculated. **(d)** Images of Hematoxylin-Eosin, Oil Red-O and Sirius red staining. Magnification 100–200X, scale bar = 200 μm. **(e)** Higher-resolution images of Sirius red. Magnification 400×, scale bar = 50 μm **(f)** Transcription factor analysis performed by the overlapping of Ingenuity Pathway Analysis predicted activation (PPA) and Opossum analysis of transcription factor binding motif enrichment analysis (MEA) in gene promoters (+/- 2kb from TSS). Arrows indicate those TF which were also found in AH patients either in early phases or in late phases of disease progression **(cfr. Fig. 2c,d),** and are colored in green when changes are consistent and in red if they are not-consistent with human data. **(g)** qPCR of liver RNA from same animals (n=3); qPCR of *HNF4*α-P1 and P2 isoforms and of *HNF4*α targets *PCK1* and *F7.* Significance was determined by two-tailed Mann–Whitney U test in b, c and g and is denoted as follows: a = $p < 0.05$, compared with control group; b = $p < 0.05$, compared with CCl4(6w) group; c = $p < 0.05$, compared with CCl4(9w) group; d = $p < 0.05$, compared with EtOH group. For box-and-whisker plots: perimeters, 25th–75th percentile; midline, median; whiskers, minimum to maximum values; individual data points are represented. The significance of DE analyses was considered when multiple testing-corrected p-value < 0.01.

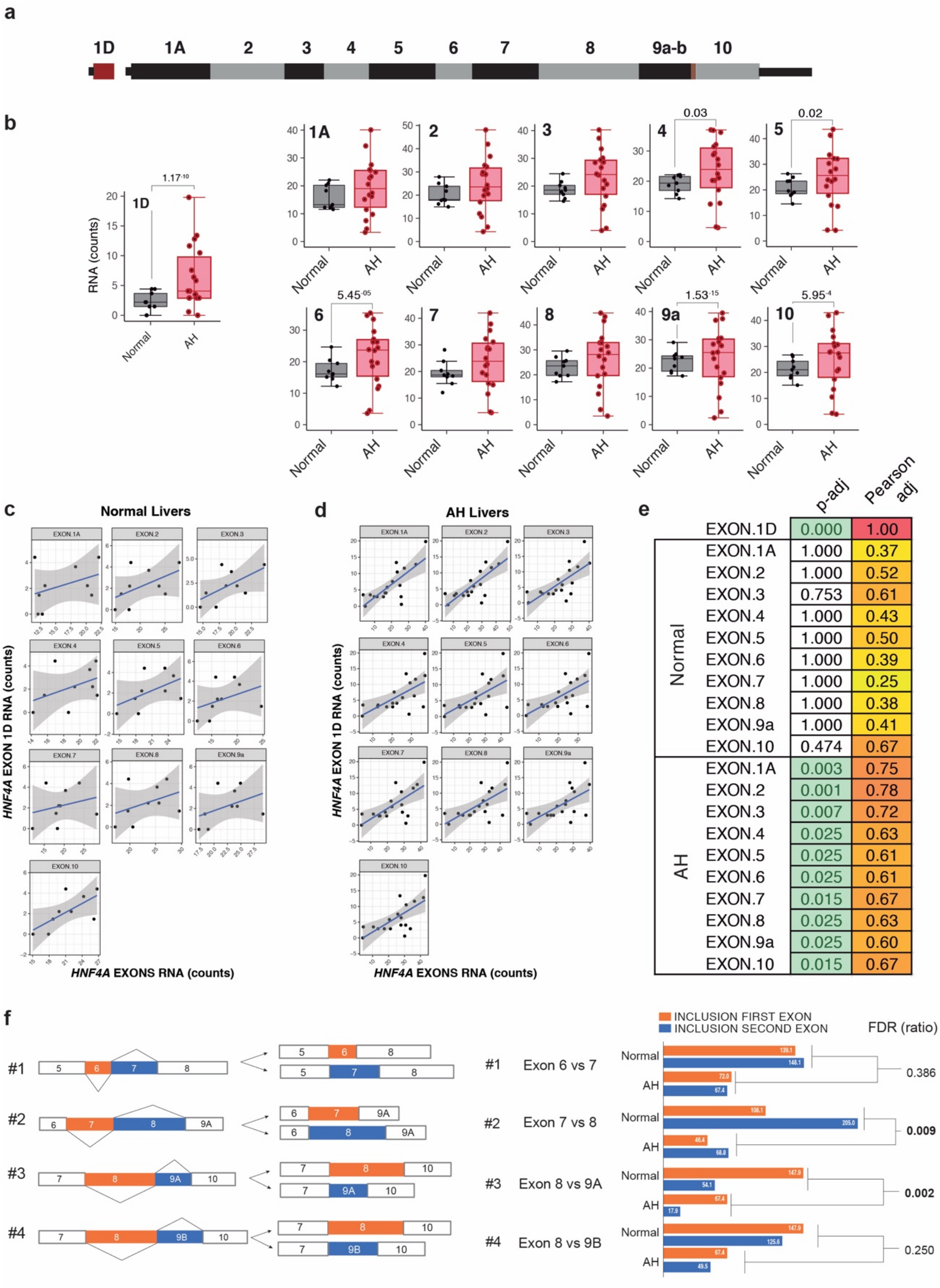

Argemi et al Supplementary Fig 5

Supplementary Fig. 5. RNA-seq analysis reveals *HNF4A* splicing dysregulation in patients with AH. (a) Scheme of exon relative size along HNF4α transcript. **(b)** Analysis of *HNF4A* exon-specific expression using TrimGalore trimming, STAR alignment and DEXSeq package. False Discovery Ratio (FDR) was calculated. **(c-e)** Pearson correlation analysis of exon 1D and each of the other *HNF4A* exons in **(c)** Normal or **(d)** AH livers. **(e)** Holm method was used to calculate adjusted p-values and Pearson's R for each correlation in (c) and (d). Green boxes (left column) indicate significant correlation (P<0.05). Red-Orange-Yellow boxes (right column) were used to illustrate R values. **(f)** Replicate multivariate analysis of transcript splicing (rMATS) pipeline was

adopted to uncover new isoforms from exon exclusion splicing events. (left) Scheme of the splicing junctions resulting in significant exon-exclusion events in Normal and AH livers. Excluded exons and possible splicing reactions are depicted as colored (orange/blue) boxes and black lines respectively. (Center) Exon sequence resulted from exon exclusion. (right) Quantification of exon-exclusion events in Normal and AH livers. FDR was calculated to detect significant differences on Exon Exclusion ratio comparing normal *vs* AH livers.

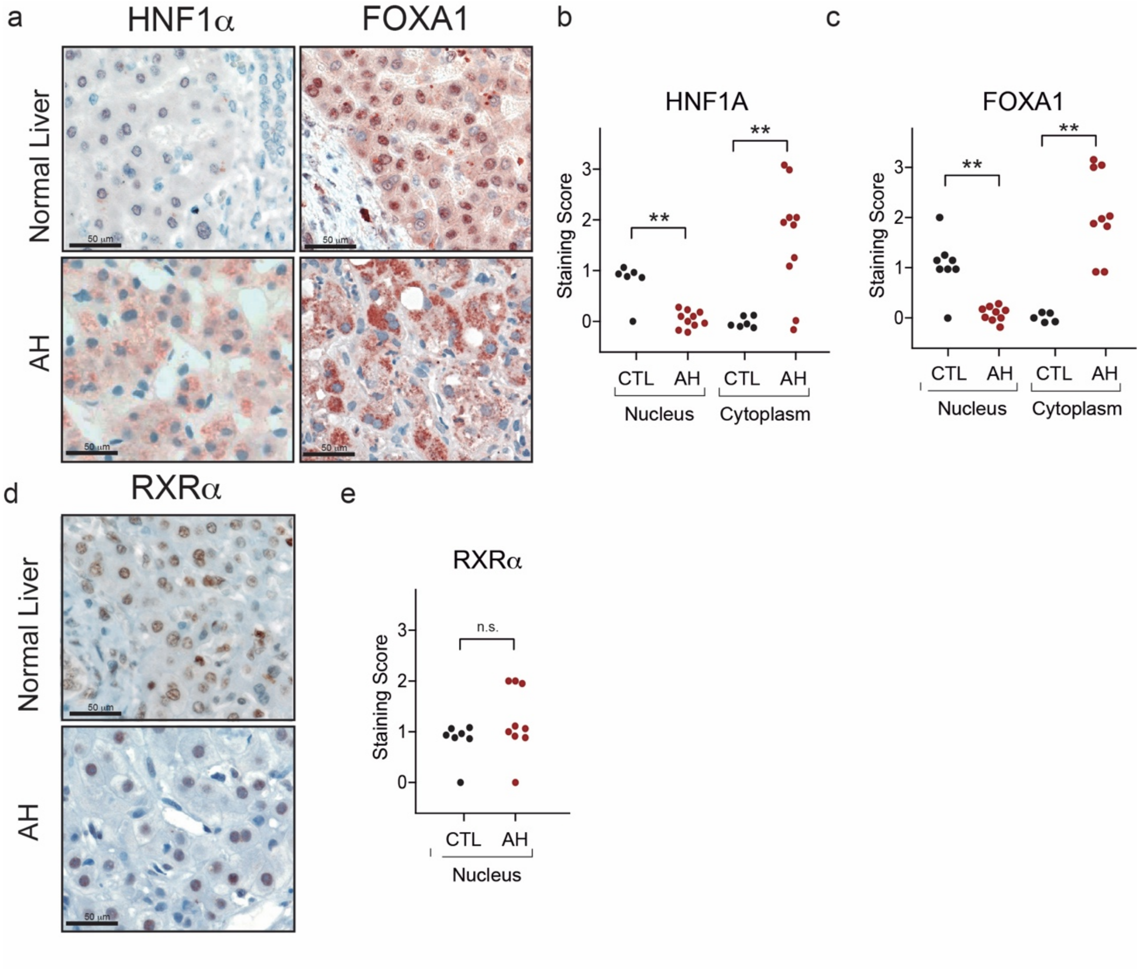

Argemi et al. Supplementary Fig. 6

Supplementary Fig. 6. Immunohistochemistry of HNF1α and FOXA1 show decreased nuclear signal and cytoplasmic staining in AH patients. (a) Representative images of HNF1α and FOXA1 Immunohistochemistry (IHC) in normal and AH livers. **(b,c)** Semi-quantitative scoring of signal intensity in the nuclei and the cytoplasm (0, low intensity/absence of signal; 1, medium intensity; 2, high intensity; 3 very high intensity) of hepatocytes for **(b)** HNF1α and **(c)** FOXA1 IHCs. **(d)** RXRα IHC in normal and AH livers. **(e)** Semi-quantitative scoring of RXRα signal intensity in the nuclei and the cytoplasm (0, low intensity/absence of signal; 1, medium intensity; 2, high intensity; 3, very high intensity). Significance of semiquantitative scoring was determined by Fisher exact probability test. *P <0.05

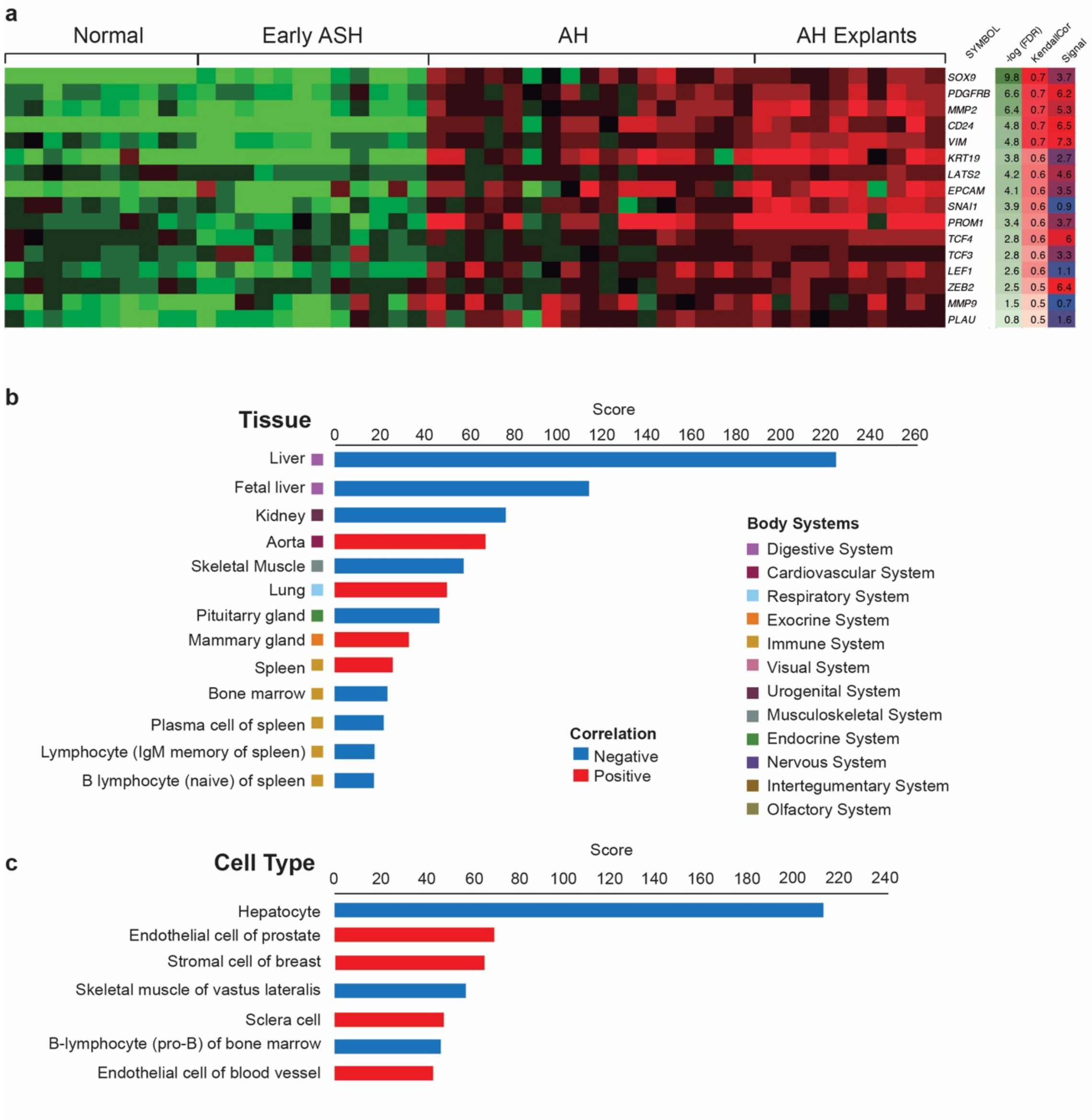

Argemi et al. Supplementary Fig. 7

Supplementary Fig. 7. Markers of hepatocyte de-differentiation and epithelial-to-mesenchymal transition (EMT) are increased in AH patients. (a) (Left-center) Heatmap showing normalized RNA-seq counts of genes related to EMT. (Right) Kendall correlation index and significance (expressed as -log of Fold Discovery Ratio (FDR) for the progression from normal to early ASH, AH and exAH. (Right) Relative transcript levels ("Signal") of each gene. **(b,c)** Results from Correlation Engine (Illumina BaseSpace) analysis of progression from normal to AH explants by correlation with specific tissue **(b)** and cell type **(c)** datasets. The score indicates the magnitude of the correlation, and the color indicates if there is a direct correlation with upregulated genes (red) or with downregulated genes (blue). Only the first hit of each organ/cell type is presented.

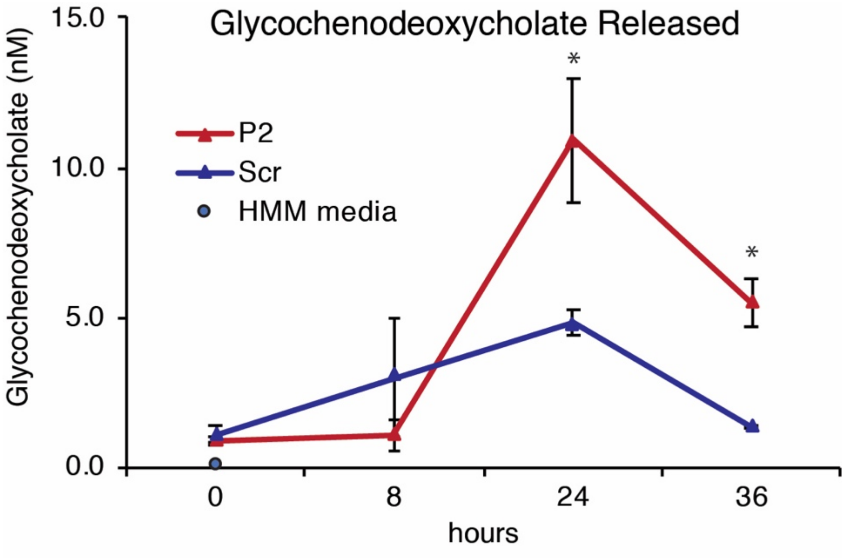

Argemi et al. Supplementary Fig. 8

Supplementary Fig. 8. Silencing of HNF4a-P2 enhances Glyco conjugation of cheno-deoxycolic acid in primary human hepatocytes. Primary Human Hepatocytes were transfected with HNF4a-P2 specific siRNA. Supernatant was collected at base line, 8h, 24h and 48h. The levels of glycochenodeoxycholate were measured by mass spectrometry. Data is presented as mean and standard error of the mean. Significance was determined by two-tailed Mann–Whitney U test *P < 0.05.

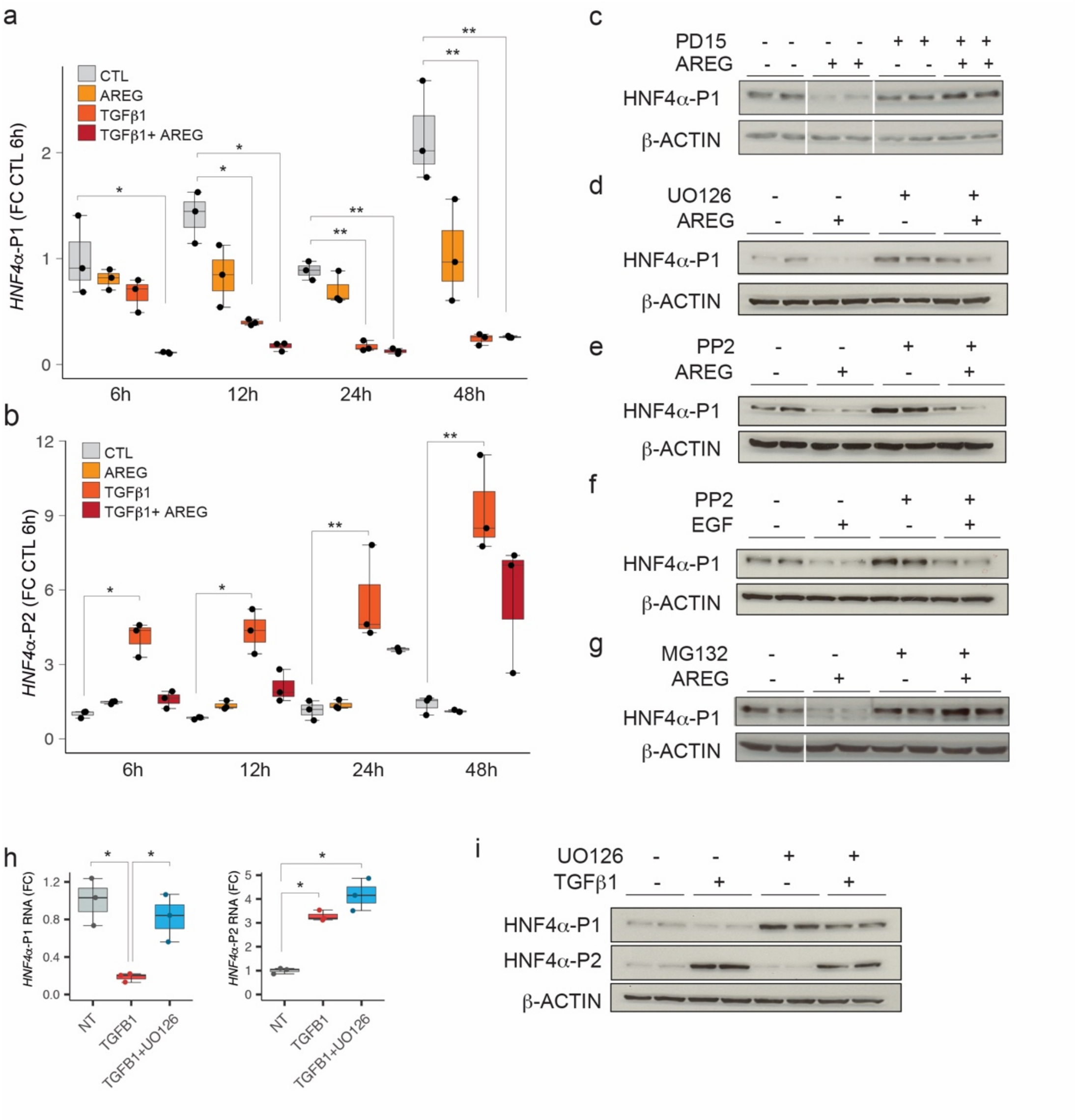

Argemi et al. Supplementary Fig. 9

Supplementary Fig. 9. Mechanism of TGFβ1 and AREG-mediated HNF4α downregulation. (a,b) Hep3B cells were serum-starved overnight before treatment with TGFβ1 (5 ng/ml) and/or AREG (50 nM) for the indicated time points (*n* = 3 for each condition). **(a)** qPCR of *HNF4*α-P1 **(b)** qPCR of *HNF4*α-P2 **(c)** Hep3B cells were pretreated with the EGFR inhibitor PD153035 (PD15, 25 μM) for 12h and then treated with AREG (50 nM) for additional 12h (*n* = 2 for each condition). Immunoblot of HNF4α-P1. **(d)** Hep3B cells were serum starved and pretreated overnight with MEK/ERK inhibitor UO126 (10 μM) and then treated with AREG (50 nM) for additional 12h (*n* = 2 for each condition). Immunoblot of HNF4α-P1. **(e,f)** Hep3B cells were serum starved and pretreated overnight with c-Src inhibitor PP2 (10 μM) and then treated with **(e)** AREG or **(f)** EGF (50 nM) for additional 12h (*n* = 2 for each condition). Immunoblot of HNF4α-P1. **(g)** Hep3B cells treated with AREG (50 nM) for 24h with the addition of proteasome inhibitor MG132 (10 μM) 2h before collection when indicated (*n* = 2 for each condition). Immunoblot of HNF4α. **(h,i)** Hep3B cells were serum starved and pretreated overnight with MEK/ERK inhibitor UO126 (10 μM) and then treated with TGFβ1 (5 ng/ml) for additional 12h (*n* = 2 for each condition); **(h)** qPCR of *HNF4*α-P1 and P2; **(i)** Immunoblot of HNF4α-P1 and P2. Significance was determined by two-tailed Mann–Whitney U test in **a** and **b**: $*P < 0.05$, $**P<0.01$. For box-and-whisker plots in **a** and **b**: perimeters, 25th–75th percentile; midline, median; whiskers, minimum to maximum values; individual data points are represented. Gene expression is presented as relative values normalized to the mean of the control.

a

Matrix ID	Name	Score	Relative score	Sequence ID	Start	End	Strand	Predicted sequence
**MA0099.2**	FOS::JUN	7.57255	0.887157893346	*HNF4A*-P2	68	74	+	TGAATGA
**MA0099.2**	FOS::JUN	6.74348	0.856895443435	*HNF4A*-P2	314	320	+	TCACTCA
**MA0099.2**	FOS::JUN	7.57255	0.887157893346	*HNF4A*-P2	341	347	+	TGAATGA
**MA0099.2**	FOS::JUN	8.77298	0.930975462764	*HNF4A*-P2	690	696	+	TGACACA
**MA0099.2**	FOS::JUN	6.65634	0.853714618169	*HNF4A*-P2	1391	1397	+	TGGCTCA
**MA0099.2**	FOS::JUN	6.69649	0.855180130106	*HNF4A*-P2	1717	1723	+	TTACTGA

b

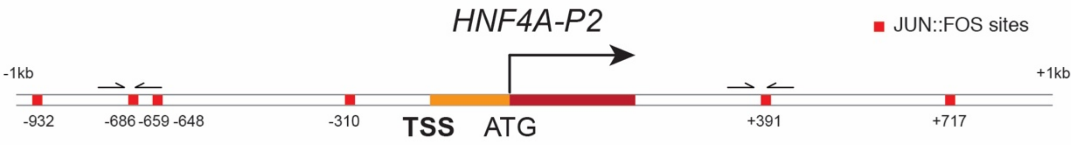

Argemi et al. Supplementary Fig. 10

Supplementary Fig. 10. c-JUN sites in HNF4a-P2 promoter region (TSS+/-1kb) (a) Output of JASPAR2018 Basic Sequence Analysis tool, using the matrix profiles "FOS::JUN" heterodimer (MA0099.2 and MA0099.3) and "JUN" (MA0488.1) **(b)** schematic presentation of HNF4a-P2 promoter region (TSS+/-1kb) with indicated the FOS::JUN sites (red squares) and the primers used for the qPCR.

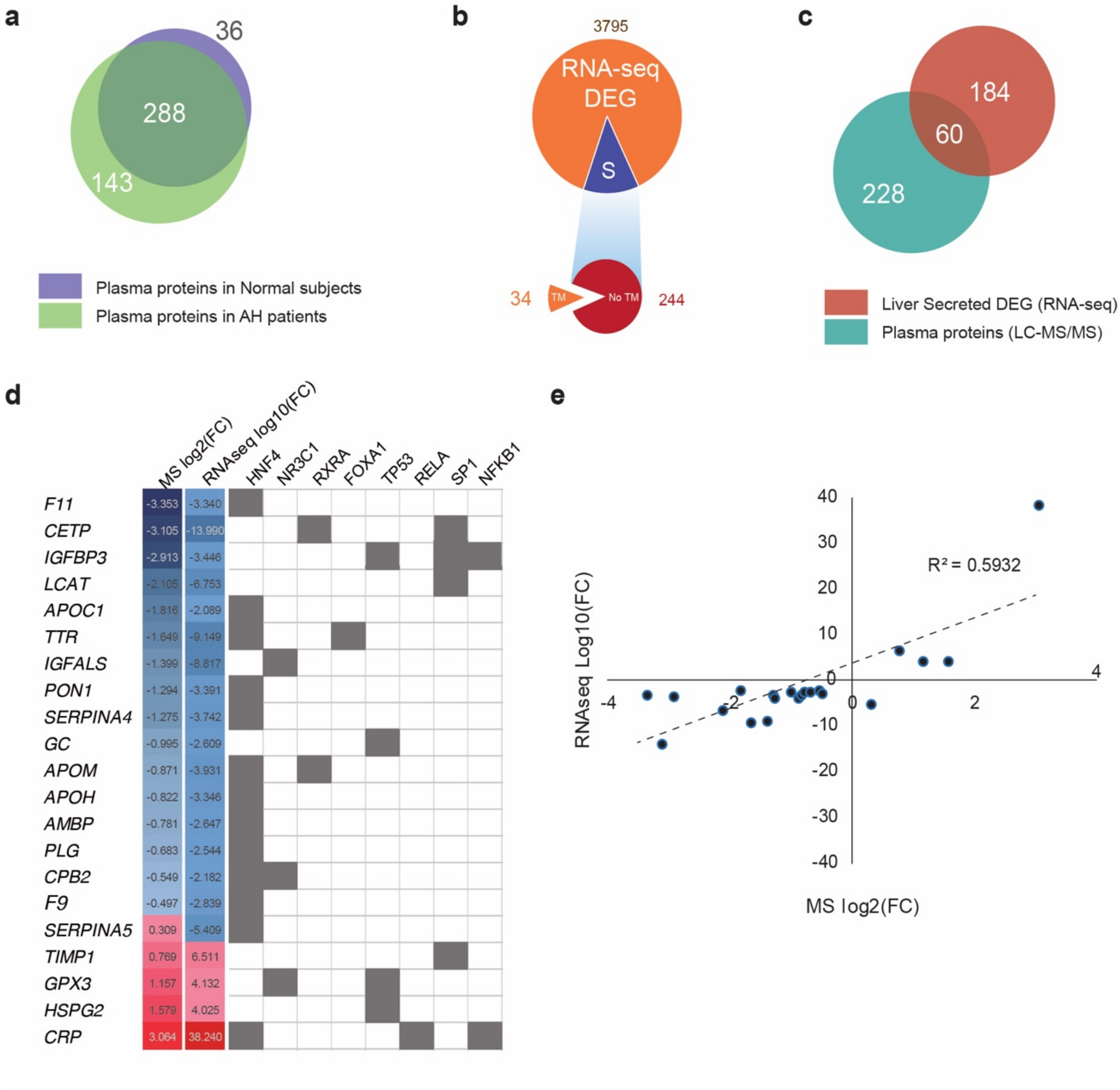

Argemi et al. Suppl Fig. 11

Supplementary Fig. 11. Plasma liver-secreted LETF-dependent protein levels correlate with liver transcript changes. Plasma from healthy controls (N=10) and from patients with AH (N=15) was pooled, depleted from top 12 most abundant proteins, digested and analyzed by liquid chromatography coupled to mass spectrometry (LC-MS/MS). **(a)** Venn diagram showing the number of common proteins detected in plasma of healthy (334 proteins) and AH (431 proteins). **(b)** Filtering of RNA-seq differential expressed genes (DEG) between Normal and AH livers, by the presence of signal peptide (278 proteins) and the absence of transmembrane domain to obtain a list of DE secreted protein coding genes (244 proteins). **(c)** Venn diagram of liver DE secreted protein coding genes and plasma protein detected by LC-MS/MS **(d)** Chart illustrating target genes of TFs predicted to inhibited in AH patients (cfr. Fig 1g) and their fold change (FC) in RNA-seq and LC-MS/MS experiments. Colors indicate relative intensity of FC in both analyses either down (blue scale) or upregulated (red scale). **(e)** Correlation plot of FC of genes in **d** in RNA-seq and LC-MS/MS experiments, indicating R^2 spearman correlation index.

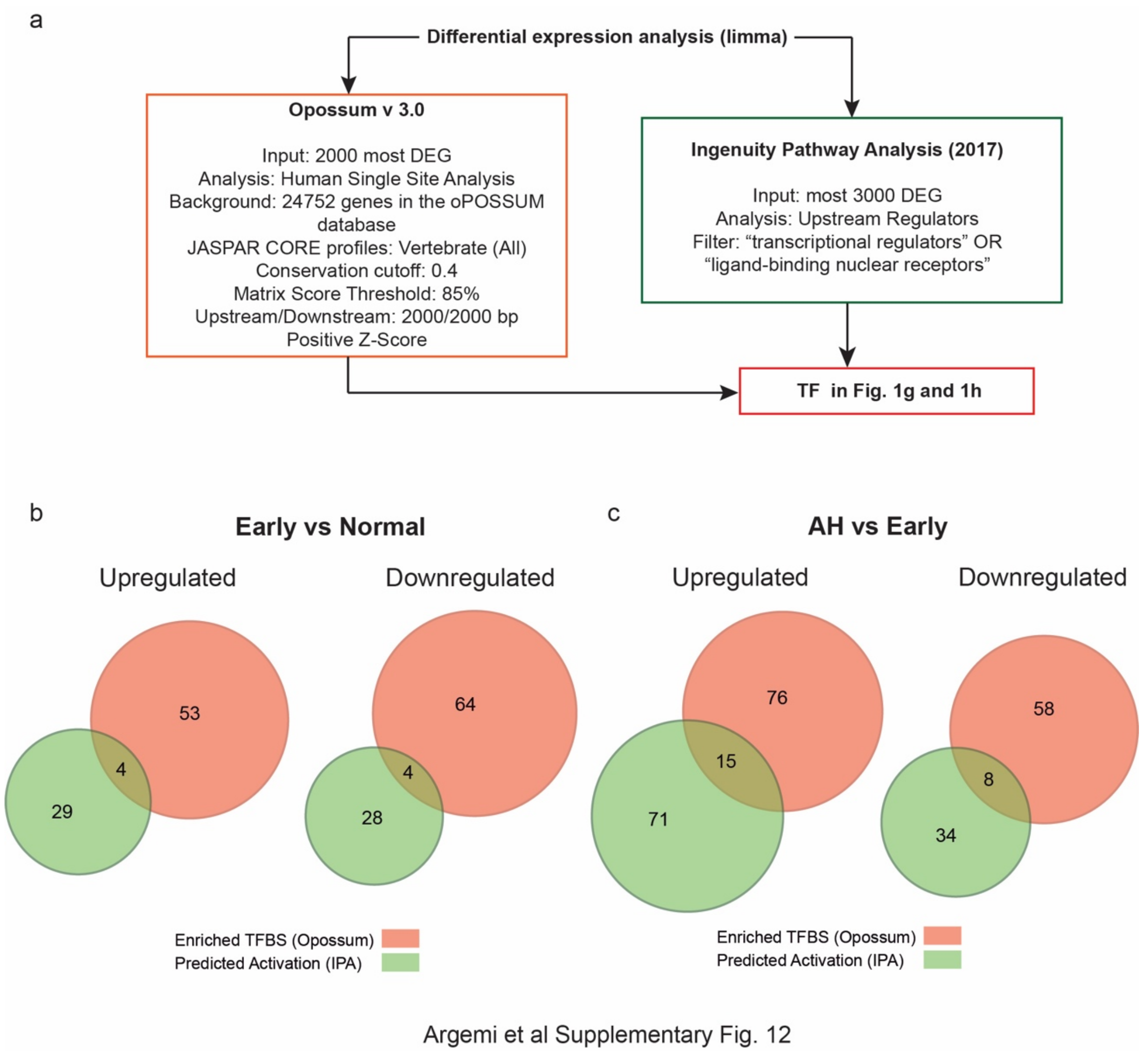

Supplementary Fig. 12. Complementary approach for identifying transcription factors that regulate liver transcriptome. (a) Scheme of unbiased complementary approach used to generate **Fig. 1g,h** and **Fig. 3p** by overlapping outputs from transcription factor binding motif searching engine (Opossum) and pathway enrichment software (Ingenuity Pathway Analysis) with the conditions used. **(b)** Venn diagram showing the overlap of Normal vs Early ASH livers or **(c)** Early ASH vs AH livers.

Uncropped Western Blots of Main Figures

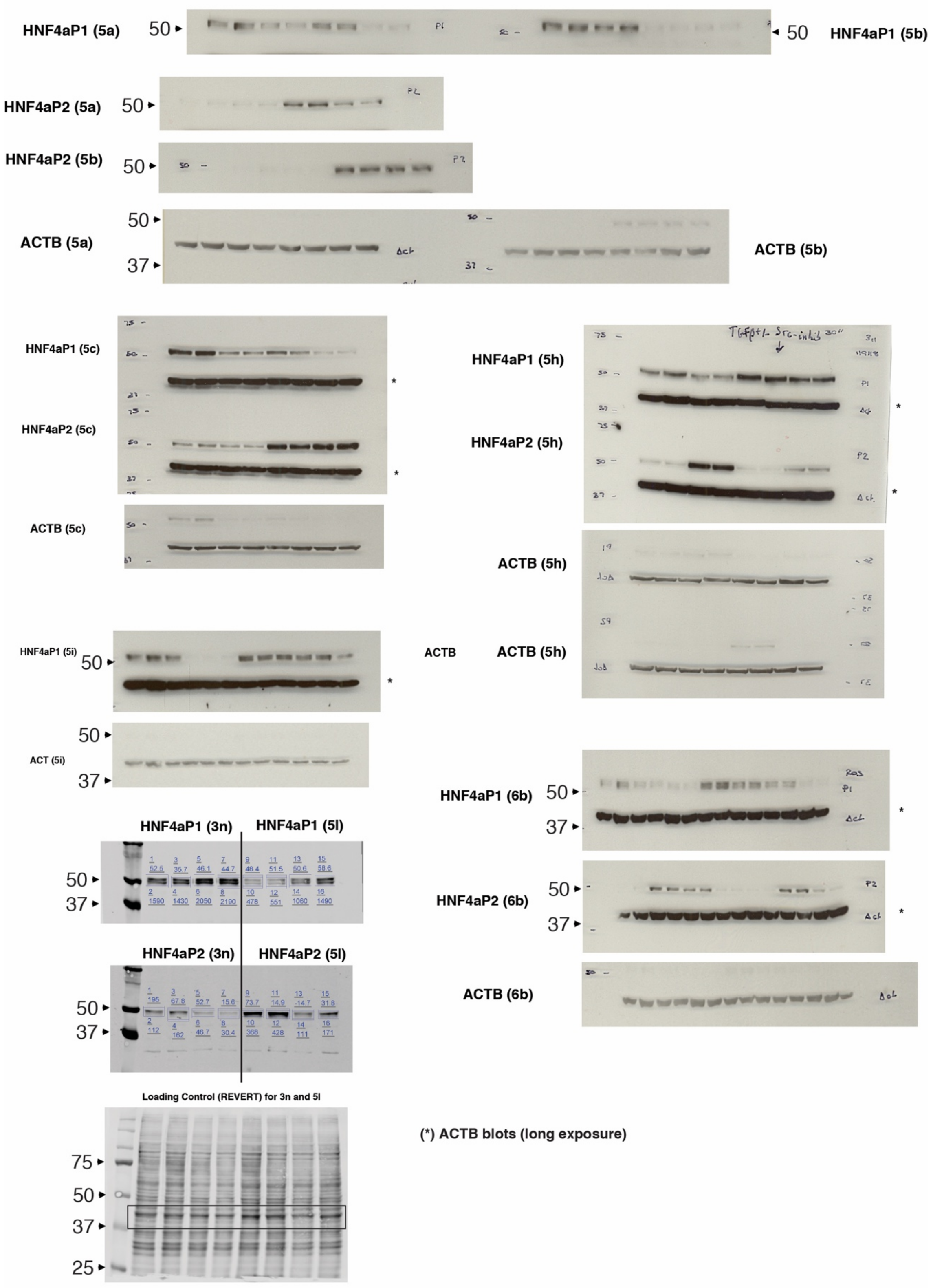

Argemi et al Supplementary Fig 13

Supplementary Table 1. Baseline characteristics of patients and controls at the time of liver biopsy.

	Early ASH n=12	Severe AH n=18	Severe AH (explants) n=11	Normal livers n=10	NASH n=9	HCV n=10	Comp. Cirrhosis n=9
**Demographics**							
Age - median (IQR)	52(48.2-58.7)	51(47.2-57.7)	48.9(48-56)	32(28.8-50.7)	49.5(43-53)	46(43-62.5)	61(51.3-66)
Gender - male n (%)	7(58.3)	11(61.1)	7(63.6)	7(70)	2(25)	5(50)	7(77.7)
**Severity scores –** median (IQR)							
Child-Pugh	N/A	11(9-11.7)	10.7(9-12)	N/A	N/A	N/A	5(5-5)
MELD	N/A	24(22-27.7)	24.5(21.4-27.2)	N/A	N/A	N/A	10(8.5-12)
ABIC	N/A	8.3(7.8-8.8)	N/D	N/A	N/A	N/A	N/D
**Decompensations** - N(%)							
Ascites	0	14(83.3)	7(72.7)	0	0	0	0
Hepatic Encephalopathy	0	5(27.8)	1(9)	0	0	0	0
Upper GI Bleeding	0	1(5.6)	2(18.2)	0	0	0	0
Acute Kidney Injury	0	10 (55.6)	N/D	0	0	0	0
Infections	0	5(33.3)	5(55.6)	0	0	0	0
**Lab parameters -** median (IQR)							
Hemoglobin g/dL	14.3(13.6-16.6)	11.6 (10.7-13.2)	N/D	14.6(12.9-15.5)	14.3(12.5-14.9)	14.8(14.2-16.5)	16.1(13.6-17.1)
WBC x109/L	5.6(4.6-7.2)	8.3 (6.6-12.7)	10.8(7.4-14.2)	5.7(5.1-7.2)	8.1(6.9-10.2)	6.4(5.9-6.8)	5.5(4.4-6.9)
Platelets x109/L	179(121-237)	118 (67-208)	N/D	237(210-282)	262(221-361)	214(174-247)	142(119-176)
AST (U/L)	107(64-154)	114.5 (62.5-158.3)	170(131-279)	21.5(18.8-26.5)	30(25-36.3)	54(41.3-63.5)	86(63-107)
ALT (U/L)	70.3(53.5-89.8)	32.5 (20-44)	N/D	25(14.8-34.3)	40(31-49.5)	85(59.8-113)	113(77.5-155)
Bilirubin mg/dL	1.2(0.7-1.5)	19 (12.3-26.7)	16.3(11.1-24.3)	0.6(0.5-0.7)	0.6(0.4-0-9)	0.75(0.6-1)	1.1(0.78-1.65)
GGT (U/L)	388(200-723)	406(165-721)	N/D	17(13.5-24.8)	27(10-31)	36.5(24.3-48.3)	45(32.7-186)
ALP (U/L)	100(62-141)	386(147-491)	N/D	147(106-191)	182(170-195)	162(134-200)	176(145-301)
Albumin (g/dL)	4.5(4.2-4.7)	2.9 (2.3-3.3)	2.4(2-3)	4.6(4.3-4.6)	4.5(4.4-4.6)	4.4(4.2-4.7)	4.1(40.3-45)
Creatinine mg/dL	0.6(0.59-0.77)	0.79 (0.61-1)	0.69(0.53-0.73)	0.8(0.74-0.9)	1.05(0.84-1.1)	1(0.98-1.1)	1(0.83-1.1)
Sodium (mEq/L)	139(136-140)	136(132-139)	N/D	140(138.7-141.5)	140(136-142.5)	142.5(140-143.3)	141(139-143)
INR	1(0.9-1)	1.6 (1.2-3.3)	1.8(1.6-2.6)	1.03(0.99-1.06)	1.19(1.06-1.35)	1.21(1.01-1.40)	1.24(1.16-1.38)

WBC: White blood count; AST: Aspartate aminotransferase; ALT: Alanine aminotransferase; GGT: Gamma Glutamyl Transpeptidase; ALP: Alkaline Phosphatase; INR: International Normalized Ratio. N/A: Do not apply; N/D: Undetermined (Missing data)

Supplementary Table 2. Oligonucleotide sequences of primers used for Real Time PCR.

Gene Symbol	Gene Name	Forward Primer (5'-3')	Reverse Primer (5'-3')
HNF4A -P1	Homo sapiens hepatocyte nuclear factor 4 alpha (HNF4A), P1 dependent isoforms (a1-6)	AGAATGTGCAGGTGTTGACG	CTCGAGGCACCGTAGTGTTT
HNF4A -P2	Homo sapiens hepatocyte nuclear factor 4 alpha (HNF4A), P1 dependent isoforms (a7-9)	GGCCATGGTCAGCGTGAACG	GCCCTTGCAGCCGTCACAGC
HNF4A-AS1	Homo sapiens HNF4A antisense RNA 1	CCTTGTTGGGGAAGCAAGTA	ACTCAGGCTGGAGTGCAGTT
PCK1	Homo sapiens Phosphoenolpyruvatecarboxykinase 1	GATGTGGCCAGGATCGAAAGCAAGAC	ATGATCCGCATGCTGGCCACCAC
ALB	Homo sapiens Albumin	TATGCCCCGGAACTCCTTTT	TGGCACACTTGAGTCTCTGT
OTC	Homo sapiens ornithine carbamoyltransferase	TTGCACTTCTGGGAGGACAT	TAGTGTTCCTGGAGCGTGAG
F7	Homo sapiens coagulation factor VII	CGATGCTGACTCCATGTGTG	GGAAGCAGGTGGGGAATAGT
BSEP (ABCB11)	Homo sapiens ATP binding cassette subfamily B member 11	CAGTGGAAAGAGGGACCCAT	TCTGCTAAAGGTCCTCGCAA
CYP2E1	Homo sapiens cytochrome P450 family 2 subfamily E member 1	GCAACCCGAGACACCATTTT	GCACACACTCGTTTTCCTGT
CYP7A1	Homo sapiens cytochrome P450 family 7 subfamily A member 1	CACCTTGAGGACGGTTCCTA	CGATCCAAAGGGCATGTAGT
CYP27A1	Homo sapiens cytochrome P450 family 27 subfamily A member 1	AGCTGCGCTTCTTCTTTCAG	GCTCCATGTCGTTCCGTACT
KRT7	Homo sapiens keratin 7	CAGGATGTGGTGGAGGACTT	AGCTCTGTCAACTCCGTCTC
EPCAM	Homo sapiens epithelial cell adhesion molecule	CAGAAGGAGATCACAACGCG	TCCAGATCCAGTTGTTCCCC
VIM	Homo sapiens Vimentin	GAGTCCACTGAGTACCGGAG	ACGAGCCATTTCCTCCTTCA
RPL4	Ribosomal Protein L4	GCTCTGGCCAGGGTGCTTTTG	ATGGCGTATCGTTTTTGGGTTGT
SMAD4	SMAD family member 4	GCTGCTGGAATTGGTGTTGATG	AGGTGTTTCTTTGATGCTCTGTCT
HNF4A Prom	TSS-809-P2	GTGTGTGAGTTTCAGCAGCA	TGAGGGGTGGAGAAACATGG
HNF4A Prom	TSS+338-P2	CTTGGTGCGAGAAGTGCTG	AGACCCCTGAGATGCATTCC
GAPDH prom	unknown	TACTAGCGGTTTTACGGGCG	TCGAACAGGAGGAGCAGAGAGCGA

Supplementary Table 3. Antibodies used for Western Blot

Protein	Supplier (#Cat)	Application	Working Dilution
HNF4α1-6 (P1 dependent isoforms)	R&D (PP-K9218-00)	Western Blot	1:1000
HNF4α7-9 (P2 dependent isoforms)	R&D (PP-H6939-00)	Western Blot	1:1000
β-ACTIN	Sigma-Aldrich (A5441)	Western Blot	1:1000
Phopho-c-JUN (T91/93/95)	Life (711207)	ChIP	5 ug/ml
RNA Polymerase II	Sigma-Aldrich (05-623B)	ChIP	1 ug/ml
Normal Mouse IgG	Sigma-Aldrich (12-371B)	ChIP	1 ug/ml

Supplementary Table 4. Antibodies and conditions for Immunohistochemistry

Antigen	Antigen retrieval<br>Incubation time<br>Buffer	Blocking<br>Incubation time	Primary antibody<br>Company<br>Dilution, incubation time	Detection System<br>Company	Chromogen
HNF4α 1-6 (P1)	Microwave, TE pH 6.0, 40’	REAL™ (Dako), 10’	Clone K9218 (Perseus), 1:100, 60‘	IDetectTM Super Stain System–HRP (Empire)	AEC
HNF4α 7-9 (P2)	Microwave, TE pH 6.0, 40’	REAL™ (Dako), 10’	Clone H6939 (Perseus), 1:100, 60’	Envision (Dako)	DAB
HNF1α	Microwave, TE pH 9.0, 40’	REAL™ (Dako), 10’	ab204306 (Abcam), 1:200, 60’	IDetectTM Super Stain System–HRP (Empire)	AEC
FOXA1	Microwave, TE pH 9.0, 40’	REAL™ (Dako), 10’	Clone #654126 (R&D), 1:100, 60’	IDetectTM Super Stain System–HRP (Empire)	AEC
RXRα	CC, 60’	REAL™ (Dako), 10’	Clone K8508 (R&D), 1:100, 32‘	Ultraview (Ventana)	DAB
