# Supplementary material for: Defective HNF4alpha-dependent gene expression as a driver of hepatocellular failure in alcoholic hepatitis
Source: Nat Commun. 2019 Jul 16;10:3126. doi: 10.1038/s41467-019-11004-3 (PMC6635373; doi:10.1038/s41467-019-11004-3)
Supplement: Supplementary file 7 — Description of Additional Supplementary Files [file 41467_2019_11004_MOESM7_ESM.pdf]

Legends for supplementary data

Title: Supplementary Data 1. STEM output tables.
Description: Short Time Expression Miner (STEM) algorithm identified 13 profiles of gene expression across the 4 selected disease stages: Normal (N) – Early (E) – Severe AH (AH)– Explants AH (exAH). In the sheet "STEM Output" the 13 profiles ordered by p-values (top) and number of genes assigned (bottom) are presented. In the sheets "Profile X", the set of genes assigned to each profile is provided, with the mean normalized expression for each gene along the disease group.

Title: Supplementary Data 2. Transcription Factor Target Genes.
Description: Results of the Transcription Factor (TF) selection for figure 2, using Opossum for scanning for transcription factor binding sites and Ingenuity Pathway Analysis (IPA) to detect upstream regulator potential. In the table, the statistics for each TF in Opossum (Z-Score and Fisher Score) and in IPA (Z-Score and p-value of overlap) are presented. In addition, for each TF, the set of differentially expressed genes included in each TF signature (in IPA) is presented.
Title: Supplementary Data 3. Genome-Wide Association Study of Transcription Factor SNPs.
Description: Results of the genomic-wide association (GWAS) analysis, focused on the TF in Figure 2. None of the Single Nucleotide Polymorphisms (SNP) reached significance. The sheets "TOP_DIRECT_SNPs" and "TOP_IMPUTED_SNPs" contain the most significant SNP within genomic regions related to TF genes. The sheet "GENE_TESTS" summarizes the statistics of SNP association analysis at a gene level.

Title: Supplementary Data 4. Top Differentially Methylated CpG.
Description: Top 1000 differentially methylated CpGs**,** presented as for output derived of DMRcate. The data include the identification of the CpG, the p-value of association to the Alcoholic Hepatitis (AH) group, its genomic coordinates and the characteristics of the Illumina Infinium-EpiC probes. It also includes the annotation of each CpG including UCSC-GeneRef, Fantom4 and Fantom5 enchancers and GencodeBasicV12.

Title: Supplementary Data 5. Analysis of SNPs in Differentially Methylated regions.
Description: A. Differentially Methylated Regions around HNF4A locus, B. SNPs located +/-1.5kb from the start and end of these DMRs were extracted from the AH GWAS dataset, C. SNPs in DM CpG loci containing HNF4A binding motifs that demonstrated a potential association with developing severe AH, D. SNPs in DMR HNF4A binding motifs-containing CpG that demonstrated a potential association with developing severe AH. For each set of SNP, the odds ratio and the upper and lower limit of the Confidence Interval (95%) are presented.
